# A systematic review of the effect of personal health records on patient activation

**DOI:** 10.1177/20552076251315295

**Published:** 2025-01-22

**Authors:** Irina Osovskaya, Ann Blandford, Henry WW Potts

**Affiliations:** 1 Institute of Health Informatics, London, UK; 2 Department of Computer Science, London, UK

**Keywords:** Personal health records, patient activation, empowerment, digital technology, computerised medical record systems

## Abstract

**Background:**

Personal health records (PHRs) or patient portals have been on the healthcare policy agenda for many countries as a promising mechanism to support patient-centred healthcare by making medical records accessible to patients and those assisting patients in health self-management. Studies on clinical outcome have been inconsistent. To help us to understand why, we propose to look at measures that precede clinical outcome, specifically patient engagement and activation. Patient activation describes the knowledge, skills and confidence a person has in managing their own health and healthcare.

**Objective:**

To systematically review the current evidence on the impact of PHRs on patient activation.

**Methods:**

A literature search was conducted for randomised controlled trials and quasi-experimental studies published up to January 2024 across EMBASE, PsycINFO, CINAHL and PubMed. Publications were included in the study if they examined any association between PHR use and activation.

**Results:**

The search initially produced 3062 papers for review, of which 88 full-text articles were screened for eligibility. Two reviewers assessed 22 of these articles, and 8 papers were identified as meeting the selection criteria. Among these, seven studies found no statistically significant differences in activation. However, one study reported a significantly greater effect than the others. Data from seven randomised controlled trials and quasi-experimental studies examining the effects of PHRs on patient activation and similar measures were extracted for meta-analysis. Overall, the use of PHRs was associated with a 0.41 standardised mean difference increase in activation (95% confidence interval 0.31–0.51). There was a high degree of heterogeneity (*I*² = 98%), with one study showing a much larger effect size compared to the rest.

**Conclusion:**

Most studies showed little impact on activation, but one study found a large effect. This study notably offered PHRs combined with health coaching and training in the use of the system to their intervention group, which may indicate an important requirement for how to get the best out of a PHR system.

## Introduction

Personal health record (PHR) implementation has been on the healthcare policy agenda for many countries.^1^ The implementation of PHRs and shared digital health systems that link patient healthcare data across multiple sources has the potential to facilitate significant advancements in healthcare efficiency, quality and performance in countries where these systems are introduced. Shared information can also support clinical research, effective public health planning and the evaluation of healthcare interventions.^2^

PHRs have become increasingly popular in recent years with many health systems implementing them to improve patient engagement and self-management. PHR implementation can be either implemented at a national level or at a health system level. Notable examples of health system implementation include MyChart,^3^ developed by Epic Systems Corporation and implemented in several health systems across the US, including the Mayor Clinic and Cleveland Clinic; Kaiser Permanente's^4^ My Health Manager system; and HealtheVet in the Veteran Health Administration in the US.^5^ A number of countries have rolled out PHRs as nationwide initiatives, including Estonia, Denmark and Australia,^6^ but there appear to be no published studies that demonstrate the impact of these systems. The UK's NHS has published a number of case studies demonstrating the benefits of PHRs.^7^

Existing PHR systematic reviews a number of systematic reviews have been conducted to critically appraise PHR interventions with relevant patient outcomes and other benefits.^1–4,15^ These studies focused on evaluating PHRs’ effects on clinical outcomes, patient activation or engagement, with most finding limited impact when PHRs were used alone. Reviews that included interventions like coaching or secure messaging noted more positive effects, particularly on engagement and clinical outcomes such as glycaemic control. Studies differed in terms of the populations studied (e.g., general patients vs. chronic illness groups), the metrics evaluated (clinical outcomes vs. patient empowerment) and the functionalities of PHR systems. Overall, heterogeneity in study design and intervention methods made it challenging to draw definitive conclusions about PHR effectiveness.

Davis Giardina et al.^8^ reviewed 27 studies (20 were randomised controlled trials [RCTs] and 7 were uncontrolled observational studies), and although glycated haemoglobin A1C (HbA1c) improved overall in 3 RCTs, the difference between the intervention and control groups was significant only in 1 trial. An observational study suggested an association between PHR use and improved laboratory values (glycated HbA1c and low-density lipoprotein cholesterol) and blood pressure (BP) and low-density lipoprotein cholesterol were not significantly different between intervention and control conditions in 1 of the aforementioned RCT. Two additional prospective studies examined the effect of PHR access on BP control in patients with chronic disease and found no impact.

Mold et al.^9^ in 2018 reviewed 28 studies (10 using surveys, 5 RCTs, 5 focus groups and interviews, 3 longitudinal cohort studies, 1 quasi-experimental and 1 interpretive review). There was a positive association between the use of PHRs and primary clinical outcome, improved glycaemic control and general care management. A number of studies in this review included PHRs that were used together with online services, such as secure messaging and electronic health reminder letters.

Han et al.^10^ reviewed 24 studies (10 RCTs, 7 quasi-experimental studies, 6 cohort studies and 1 mixed method study) and found that the effects of PHRs on clinical outcomes including BP, glucose, cholesterol and weight loss were mixed. For example, of five studies in which BP was included as an outcome, only one found improved BP control. Similarly, less than half of the seven studies including glucose control as an outcome had a significant finding and this was either in a non-controlled setting with no comparison group or only for a short term (6 months). Effects of cholesterol control were also overall insignificant—only one of five studies had significant reduction in low-density lipoprotein.

de Lusignan et al.^11^ in 2014 systematically reviewed 143 studies and identified several primary findings related to patients’ online access to their PHRs. Online access to health records can lead to improved patient outcomes such as better medications adherence, increased patient satisfaction and better communication with healthcare providers.

Ammenwerth et al.^12^ in 2021 systematically reviewed 10 randomised control studies and cluster-randomised trails and found low quality evidence suggesting no or little effect on HbA1c level, no or little difference in systolic or diastolic BP with the intervention, little or no effect on BMI or weight and no effect on 10-year Framingham risk score.

In summary, evidence suggests that PHR interventions may have little or no effect on clinical outcomes compared with usual care. In order to understand the reasons behind the limited effects of PHRs on patient clinical outcome, it is useful to consider a purported mechanism of action, namely patient activation and engagement.

Healthcare bodies are interested in increasing patient activation.^13^ Patient activation is a widely recognised concept that describes the knowledge, skills and confidence a person has in managing their own health and healthcare. The Patient Activation Measure (PAM) is the most commonly used measure of activation.^14^ PAM is endorsed by NHS England as a validated measure of the concept. Evidence shows that when people are supported to become more activated, they benefit from better health outcomes, improved experiences of care and fewer unplanned care admissions.^15^

According to Fumagalli et al.,^16^ empowerment represents the possession of conditions that make patients ‘willing and able’ to play an active role in their care. Activation and empowerment interconnect as concepts and relate to an increased ability, motivation and growing patient awareness of having an important role in the management of their own healthcare.^12^ The terms ‘patient empowerment’, ‘patient activation’ and ‘patient engagement’ are frequently used in digital health and often interchangeably. Digital health practitioners^17^ and researchers^16^ have attempted to clarify boundaries, connections and intersection. In the Rislings’ model,^17^ empowerment, activation and engagement are three stages in a process of how a user engages with a technology. Empowerment is how much control is put in the hands of the patient. Activation is a measure of how patients are getting through the early days of an intervention. If the two stages have gone well, this will result in user engagement—ongoing, demonstrated interaction with the technology that presumably will result in improved health outcomes.

Using PHRs may help patients take an active role in their healthcare^18^ as a result of an improved understanding of their health, related conditions and treatment.^19^ However, there is still a lack of understanding about how best to leverage PHRs to engage patients in their own healthcare and what the impact of these tools can be on patient engagement and activation. We seek to review the evidence on whether access to PHRs impacts positively on patient activation by providing better access to one's own health data. This systematic review aims to explore the evidence that supports or contradicts the hypothesis that PHRs increase patient activation.

There are diverse approaches possible to studying digital health interventions.^21^ For this review, it was decided to use just RCTs and quasi-experimental studies. RCTs are considered the gold standard for testing causal relationships between an intervention and an outcome.^22^ Quasi-experimental studies are useful when RCTs are not feasible or ethical, such as when the intervention is already in place or when it is not possible to randomly assign participants to groups. Despite the limitations of quasi-experimental studies, they can still provide valuable insights into the effectiveness of interventions when conducted properly.^23^

## Methods

### Search strategy

A search was conducted in EMBASE, PsycINFO, CINAHL and PubMed (up to December 2021).

The following terms were searched in free text or key words: ((personal* health data) OR (Web-based Personal Consumer Health Record*) OR (personally controlled health record*) OR (personal health record*) OR (PCHR*) OR (PHR) OR (PHRs) OR (patient portal)) AND ((patient engagement) OR (patient activation) OR (patient empowerment)). These terms were chosen as we have noted patient portal synonyms to describe ways to give patient access to their own health data. We picked generic language for outcomes. Although PAM is commonly used, we also recognised that the terms patient activation can be used without necessarily applying a PAM measure tool.

### Eligibility and inclusion

Inclusion criteria were studies with an element of primary data collection; using RCT and quasi-experimental study designs; and an outcome of patient activation, including PAM or similar.

Full-text articles were screened for eligibility and rejected papers were randomly crossed-checked. Possible papers for inclusion were reviewed by two reviewers to identify those fully meeting selection criteria. Any discrepancies were resolved through discussion.

Data extracted included author, year, country of study, study design, study duration, population, selection criteria, sample size, age in years, intervention type, description of intervention, outcome measures, control group treatment and effect size/impact/findings.

### Quality appraisal

Data quality was appraised using the Joanna Briggs Institute (JBI) appraisal checklists for RCTs^24^ and quasi-experimental studies.^25^ The JBI checklists composed of 13 questions for RCTs and 9 for quasi-experimental studies. Studies were rated 1 if they included a component of the quality rating and 0 if they did not identify and each study total score is calculated (Tables 1 and 2).

**Table 1.** RCTs: JBI checklist and scores of included studies.

Item	Earnest et al.^27^	Tuil et al.^20^	Wagner et al.^29^	Druss et al.^32^	Creber et al.^33^	Carroll et al.^34^
1. Was true randomisation used for assignment of participants to treatment groups?	0	1	1	1	1	1
2. Was allocation to treatment groups concealed?	0	0	0	0	0	1
3. Were treatment groups similar at the baseline?	1	1	1	1	1	1
4 Were participants blind to treatment assignment?	0	0	0	0	0	0
5. Were those delivering treatment blind to treatment assignment?	0	0	0	0	0	0
6. Were outcomes assessors blind to treatment assignment?	0	0	0	0	0	0
7. Were treatment groups treated identically other than the intervention of interest?	1	1	1	1	1	1
8. Was follow up complete and if not, were differences between groups in terms of their follow up adequately described and analysed?	0	1	1	1	1	1
9. Were participants analysed in the groups to which they were randomised?	1	1	1	1	1	1
10. Were outcomes measured in the same way for treatment groups?	1	1	1	1	1	1
11. Were outcomes measured in a reliable way?	1	0	1	1	1	1
12. Was appropriate statistical analysis used?	1	1	1	1	1	1
13. Was the trial design appropriate, and any deviations from the standard RCT design (individual randomisation, parallel groups) accounted for in the conduct and analysis of the trial?	1	1	1	1	1	1
**Total score**	**6**	**8**	**9**	**9**	**9**	**10**

1: yes/good design; 0: no/bad design.

**Table 2.** Quasi-experimental studies: JBI checklist and scores of included studies.

Item	Riippa et al.^23^	O’Leary et al.^31^
1. Is it clear in the study what is the ‘cause’ and what is the ‘effect’ (i.e., there is no confusion about which variable comes first)?	1	1
2. Were the participants included in any comparisons similar?	1	1
3. Were the participants included in any comparisons receiving similar treatment/care, other than the exposure or intervention of interest?	1	1
4. Was there a control group?	1	1
5. Were there multiple measurements of the outcome both pre and post the intervention/exposure?	1	0
6. Was follow up complete and if not, were differences between groups in terms of their follow up adequately described and analysed?	1	1
7. Were the outcomes of participants included in any comparisons measured in the same way?	1	1
8. Were outcomes measured in a reliable way?	1	1
9. Was appropriate statistical analysis used?	1	1
**Total score**	**9**	**8**

1: yes/good design; 0: no/bad design.

Each article was reviewed by one reviewer (IO) and checked by a second reviewer (HP). No articles were excluded on the basis of their review and score.

### Screening

**Figure 1.**
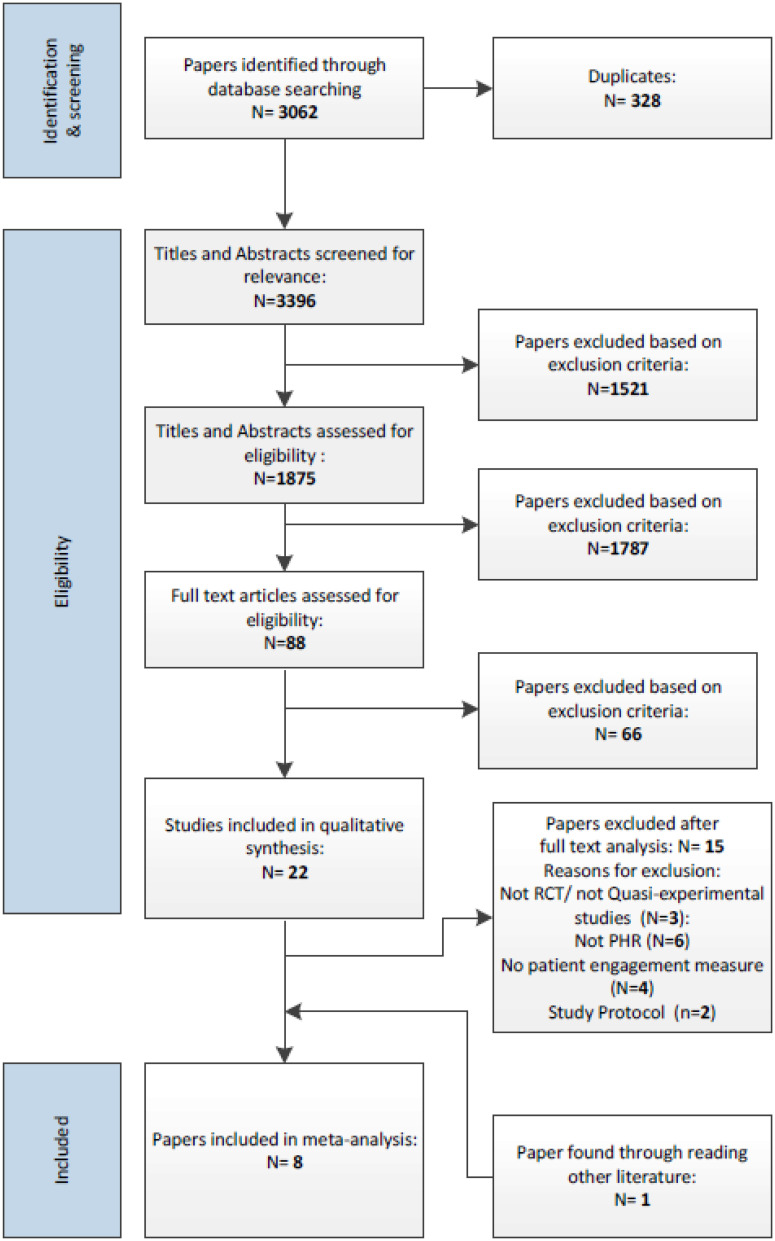
Flow diagram showing article selection strategy in PRISMA format.^26^

Studies were selected in line with the process shown in Figure 1.

## Results

### Description of included studies

The search produced 3062 papers for initial review. 88 full texts were read. Eight studies met the inclusion criteria: Earnest et al.^27^; Tuil et al.^28^; Wagner et al.^29^; Riippa et al.^30^; O’Leary et al.^31^; Druss et al.^32^; Creber et al.^33^; and Carroll et al..^34^ These are summarised below and in Table 3.

**Table 3.** Characteristics of included studies.

Author, year	Country	Study design	Study duration	Population/selection criteria	Sample size	Age in years, mean (SD)	Intervention type	Description of intervention	Outcome measures	Control group treatment	Effect size/impact/findings
Earnest et al.^27^	US	RCT	12 months	394 patients in the practice, 107 enrolled in the study, 54 assigned randomly to the intervention group	107	Mean 54 (control), 58 (intervention)	PHR	SPPARO (System Providing Patients Access to Records Online) is a secure Web interface to three components: medical record (echocardiogram, lab and test reports), a guide to heart failure and a messaging system. The clinical notes were dictated by medical providers and transcribed after every office visit.	Quantitative and qualitative techniques were used to answer the following questions: 1. How was SPPARO used in practice? What functions were used most frequently? Which patients used the system most frequently? What was it like to use the system? 2. What are the attitudes of patients and physicians towards patient-accessible medical records, and how do they differ? How do naive and experienced attitudes differ? Also, validated questionnaires were used: KCCQ, Kansas City Cardiomyopathy Questionnaire, PES	Standard care	In questionnaires and interviews, patients were significantly more likely than physicians to anticipate benefits of SPPARO and less likely to anticipate problems. Attitudes of subjects did not diverge from controls after the intervention period. In post-trial focus groups, SPPARO users described its practical benefits. Comprehending medical jargon was a minor obstacle. Physicians anticipated that implementing SPPARO might increase their workload and distort their clinical interactions. In post-trial interviews, physicians and staff reported no change in their workload and no adverse consequences. All of the physicians ultimately supported the concept of giving patients online access to their clinical notes and test results.
Tuil et al.^28^	Netherlands	RCT	12 months	199 patients scheduled for their IVF or ICSI treatment between Jan 2004 and July 2004 were informed about the study. Patients who returned their signed consent forms were randomised.	122	Male**:** Research 36.04 (±5.6), Control 36.92 (±5.8) Female**:** Research 32.85 (±3.8) Control 32.56 (±3.1)	Website connected PHRs	Website for patients who were undergoing IVF treatment with access to their medical records. The website aids patients in interpreting the medical data and also enables online communication between patients and their physicians. It provides patients with access to their own medical records, available information concerning their IVF or ICSI treatment and offers a number of communication options, inc email function, a discussion forum, and a chat room. Physicians actively participate in the latter two to moderate the discussions, to answer the patients questions, and to correct any incorrect information.	The **main outcome** measure was patient empowerment - multidimensional concept composed of 1) self-efficacy, 2) actual and perceived knowledge, and patients’ involvement in the decision process. Self-efficacy was measured by using the General Self-efficacy Scale, as well as an IVF specific self-efficacy measure. The patients’ objective knowledge about the IVF treatment was assessed by using the sum score of 10 multiple-choice questions concerning 10 key moments in an IVF treatment. These **secondary outcome** measures were 1) patient satisfaction (measured by the patient satisfaction questionnaire); 2) the meaning of fertility problems, established with use of the Illness Cognition Questionnaire, which resulted in subscales for helplessness and acceptance; social support, (assessed through the Inventory for Social Support); 3) state anxiety (measured with the State-Trait Anxiety Inventory); and, 4) depression (which we measured through the Beck Depression Index for Primary Care)		Statistical analysis was carried out by using repeated measures multivariate analysis of variance (MANOVA) to determine the effect of the PHR on the multidimensional concept of patient empowerment. empowerment. This analysis showed **no significant interaction effect** (group by time) on the combined dimensions of patient empowerment (general self-efficacy, IVF-specific self-efficacy, subjective knowledge, objective knowledge, and involvement in the decision process): Hotelling-Lawley trace ¼ 0.025; *F*(5, 160) ¼ 0.387; P¼ 0.857 for female participants; Hotelling-Lawley trace ¼ 0.009; *F*(5, 160) ¼ 0.145; P¼ 0.981 for male participants. Moreover, univariate analysis (using repeated measures ANOVA) of the individual constituting factors of patient empowerment did not show significant differences in per-person changes between the two groups
Wagner et al.^29^	US	RCT		1686 participants assessed for eligibility, 446 met inclusion criteria, 194 of those received PHR and 252 allocated to care as usual. 193 with PHR remained in the study (data analysed) and 250 with care as usual	443	Mean 55	**PHR**	The Cerner Corporate proprietary PHR system (IQHealth) deployed under the brand name MyHealthLink. This PHR is tethers to to EMR and allows view only into PHR. MyHealthLink allows secure messaging, access to educational materials, medication interaction checking, recording health measures, viewing some data and goal setting/health diary.	BP tracking; Patient Activation Measure by **PAM- SF (short form)** and **PEM (**PES**);** Quality of care assessed by assessed by using the Clinical and Group Assessment Score (**CAHPS) and Patient assessment of Chronic Illness Care.** Frequency of use of medical services was self-reported.	Standard care	Among those patients provided the PHR, utilisation of PHR was quite low with only 26% using it frequently (twice a month). Minimal difference between patients provided with PHR access and those without. Access to PHR failed to activate patients (PAM activation scores 71.4 compared with 69.1), provide outcomes, increase satisfaction with care or change frequency of use of medical devices.
Druss et al.^32^	US	RCT	12 months	644 participants were assessed for eligibility -patients with schizophrenia, bipolar disorder, posttraumatic stress and major depression and one or more medical condition were eligible to take part. Participants were also required to have primary care provider and to have a minimum of a six-grade reading level. All 199 patients scheduled for their IVF or ICSI treatment between Jan 2004 and July 2004 were informed about the study. Patients who returned their signed consent forms were randomised.	170	Mean 49.3 years (SD = 7.62)	**PHR**	MyHeath record is an adaptation of the Share Care Plan. Core feature inc personal details, diagnosis, goals, health indicators inc cholesterol and glucose levels, medications, allergies, immunisations, health and family history.	The **primary study outcome** was quality of medical inc 1) quality of preventative services and 2) quality of cardiometabolic care. **Secondary outcome** inc: 1) health services us, inc mental and medical impatient/outpatient and emergency department use 2) patient action as measures by PAM- 19	Standard care	Both groups exhibited small improvements in patient activation – 12.5% (intervention) and 15.7% (control).
O’Leary^31^	US	Quasi- experimental		283	202	Intervention-unit patients’ mean (SD) age was younger at 46.7 years (16.7) vs 51.4 years (17.3); P¼ 0.05	**PHR/patient portal**	Mobile patient portal application including pictures, names, and role descriptions of team members, scheduled tests and procedures, and a list of active medications	**PAM- SF** (short form) Use and satisfaction with the patient portal application Knowledge of team members, roles, and aspects of cares ** **		The mean (SD) PAM-SF score was higher for intervention-unit patients, but the result was not statistically significant at 64.1 (13.4) vs 62.7 (12.8); P¼ 0.46. Similarly, the PAM-SF was not significantly higher in multivariable analyses controlling for patient age (b¼ 1.58; P¼ 0.41).
Riippa et al.^30^	Finland	Quasi-experimental	6 months	Chronically ill patients from public primary care receiving immediate access to a patient portal. At least 2 treatable health conditions assessed by HCPs, bank identifiers, access to Internet, willing and able (according to HCP) to engage in the portal. Intervention: patients with odd dates birthday; control: even.	137 patients (80 patients in the intervention group and 57 patients in the control group	At least 18 years of age	**Patient portal**	1) the patient's own patient records, provided and maintained by the healthcare provider with diagnoses of chronic illnesses and permanent medication prescriptions, 2) laboratory results with statements from a healthcare professional, 3) vaccination history, and 4) electronic messaging with a healthcare professional. The names of diagnoses, medicines, and laboratory results were linked to relevant additional information in the online medical information service, Health Library	Incremental direct heath care costs, health status based on the Short-Form Health Survey, version 2 (SF-36v2), and patient activation based on the short form of the Patient Activation Measure (**PAM-13**) were compared to standard care in a 6-month follow-up.	Patient receiving standard care, received access to portal 6 month later	The mean change in the patients’ activation score (**PAM-13**) was 2.8 points (95% CI −2.2 to 7.8) higher in the intervention group, compared to the control group in the adjusted sample, and 2.6 points (95% CI −1.8 to 7.1) higher in the intervention group, compared to the control group in the unadjusted sample. The proportion of patients with clinically meaningful change (≥5 points) in patient activation was 7.0% higher in the intervention group in the matched sample and 5.7% higher in the intervention group in the unmatched sample. Differences in patient-reported physical and mental health changes were minor and changed sign from the matched (physical health, mean 1.2, 95% CI −3.3 to 5.7; mental health, mean 0.8, 95% CI −3.6 to 5.2) to the unmatched sample (physical health, mean −0.4, 95% CI −4.7 to 3.9; mental health, mean -0.4, 95% CI −4.8 to 4.0). The effect of the portal access on cost of care was ambiguous, changing from more to less costly depending on the model used
Creber et al.^33^	US	RCT	39 months	English- or Spanish-speaking patients from 2 cardiac medical-surgical units at an urban academic medical centre All newly admitted patients were screened for eligibility and approached if not already enrolled. Patients in contact isolation rooms or patients who have been admitted for over 2 weeks from this index hospitalisation were not eligible for enrolment. All English- or Spanish-speaking adult patients aged 18 years or older were eligible for the study.	426 patients randomised into to 1 of 3 groups: 1) usual care, 2) tablet with general internet access (tablet-only), and 3) tablet with an inpatient portal	59.2 ± 16.0 (*p* = 0.001)	**Patient portal**	The portal contained the patient's clinical information updated directly from the hospital's EHR (Allscripts Sunrise, Allscripts Corp., Chicago, IL) every 15 min. The portal's features included: 1) names & photos of care team members, 2) medications being administered, 3) short videos explaining the purpose of each medication as well as potential side effects, 4) links to comprehensive medication information from MedlinePlus, 5) documented allergies, 6) diagnostic test orders & results, 7) current documented diet, 8) vital signs and weight, 9) functionality to report pain level,10) functionality to communicate comments and questions to care team members, and 11) functionality to acknowledge care team members with a star rating.	The primary study outcome was patient activation, measured with the 13-item Patient Activation Measure (**PAM-13**). The secondary outcomes included all-cause hospital readmissions to academic medical centre within 30 days, length of hospital stay, engagement with personal health information, measured using questions derived from the Telemedicine Satisfaction and Usefulness Questionnaire, and patient satisfaction.	Patient receiving standard care	There were no significant differences in patient activation scores among the 3 groups in unadjusted (P¼ 0.392) or adjusted (P¼ 0.418) analysis. Almost half of participants (45.6%) had a minimum change in PAM-13 score by 3.8614.4 points over the course of the hospitalisation, which is considered a clinically significant improvement in patient activation. 48 Participants in the portal group who used the portal more frequently (2 or more total logins) also did not have higher activation (P¼ 0.110). There were significant differences in the readmission rate among 3 groups [5.5% (portal), 12.9% (tablet-only), and 13.5% (usual care), P¼ 0.044]. Across all 3 groups, patients in the portal group were more likely to report that the care team uses information that patients provide to them [4.660.6 (portal) vs. 4.260.9 (tablet only) and 4.460.7 (usual care); *p* < 0.001]. Overall, patients were highly satisfied with their healthcare and healthcare providers, and there were no differences among the 3 groups.
Carroll et al.^34^	US	RCT	35 months	Patients with confirmed HIV diagnosis, age ≥ 18 years, and receipt of HIV/primary care at a participating site. Exclusion criteria included, prior participation in the pilot, the inability to provide informed consent or limited English proficiency.	360	Intervention: 51.7 (10.7) Control: 51.2 (11.3) *p*-value 0.69	**ePHR** ** **	**Tech:** Apple iPod touch with customised ePHR. Key features included 1) drop down menus for common HIV medications with accompanying pill pictures; 2) common lab tests with brief explanations; 3) ability to set reminders for appointments, as well as for taking and refilling medications; 4) personalised “prompt list” of potential questions for the patient to ask their clinician. **Intervention** consisted of six 90-min training sessions, in groups of mean participant size *N* = 11 (range 7– 15), co-facilitated by staff coaches and trained peer educators. The sessions focused on basic HIV literacy, basic eHealth competency and use of the ePHR,. Participants were encouraged to assist each other in learning and to celebrate successes. After completing the group training sessions, each patient received one 20–30 min individual coaching session. A staff coach met with each patient before the patient's next HIV office visit to reinforce skills.	The primary study outcome was patient activation, measured with the 13-item Patient Activation Measure (**PAM-13**). Secondary measures included eHealth literacy (eHEALS), Decision Self-efficacy (DSES), Perceived Involvement in Care Scale (PICS), health (SF-12), receipt of HIV-related care, and change in HIV viral load (VL)	Usual care plus iPod	The intervention group showed significantly greater improvement than the control group in the primary outcome, the PAM (difference 2.82: 95% confidence interval [CI] 0.32–5.32). Effects were largest among participants with lowest quartile PAM at baseline (*p* < 0.05). The intervention doubled the odds of improving one level on the PAM (odds ratio 1.96; 95% CI 1.16–3.31). The intervention group also had significantly greater improvement in eHEALS (difference 2.67: 95% CI 1.38–3.9) and PICS (1.27: 95% CI 0.41–2.13) than the control group. Intervention effects were similar by race/ethnicity and low education with the exception of eHealth literacy where the effects were stronger for minority participants. No statistically significant effects were observed for decision self-efficacy, health status, adherence, receipt of HIV relevant care, or HIV viral load.

A final total of 8 papers were subject to full data extraction. Through quality appraisal, we identified strengths and weaknesses in studies’ methodology, but the overall analysis and conclusions have not been affected.

Six of the eight included studies were based in the US and remaining two in Europe (the Netherlands and Finland).

Of the US studies, only two were based in hospitals and the remainder in primary care and specialist clinics. Patients across eight studies had long-term health conditions ranging from serious mental health disorder to hypertension, long-term heart condition, HIV and undergoing IVF. Only one study^31^ had a mixed range of patient conditions from acute, such as injury and poisoning, to a wide variety of chronic conditions.

Carroll et al. found a large, significant effect that stands out compared to the other studies. Six of the other seven studies did not find any statistically significant differences in activation and patient empowerment measures. Ernst et al., Druss et al. and Creber et al. conclude PHRs are beneficial, but based on other outcome measures in their studies. Riipa et al. and Tuil et al. focused on activation or empowerment and report non-significant results but still offered optimistic conclusions in favour of PHRs. In contrast, O’Leary et al. conclude that PHRs do not make a difference to activation. Wagner et al., with a non-significant result on patient activation, are equally pessimistic, although they do report a significant improvement on a separate patient empowerment score.

Earnest et al.^27^ evaluated the experiences of patients and physicians using System Providing Patients Access to Records Online (SPPARO) in the US. No statistically significant differences were found between groups, but the Patient Empowerment Score declined for patients as a whole.

Tuil et al.^28^ evaluated an Internet-based PHR for patients undergoing IVF treatment in the Netherlands. The PHR users were relatively young and well-motivated patients, who expressed the explicit need for Internet-accessible medical records. The authors viewed the concept of patient empowerment as having conflicting definitions and connecting concepts such as self-efficacy, knowledge about disease and treatment and patients’ involvement in decision-making process. They thus used as the main outcome measure a questionnaire that consisted of items about self-efficacy, actual and perceived knowledge, and involvement in the decision process. The study found no statistically significant differences between an experimental and a control group on their various measures of empowerment (*p*s > 0.4).

Wagner et al.^29^ conducted a cluster randomised effectiveness trial with 24 primary care physicians recruited from one Family Medicine and one from Internal Medicine ambulatory clinics at a tertiary academic medical centre in the US. Physicians were randomised into two groups: intervention (PHR) or control (care as usual). This study employed the Centre Corporation proprietary PHR system (IQHealth) deployed under the brand name MyHealthLink. PAM scores were not significantly different between the experimental and control groups (*p *= 0.23). However, Wagner et al. did show a significant difference on a related outcome, the Patient Empowerment Scale (PES; *p *= 0.019). The PES assesses patient's perceived risks and benefits of access to their own health information and, similar to PAM, assesses patients’ knowledge about their condition and better understand their doctor's instructions. Few patients provided with a PHR actually used the PHR with any frequency. The authors concluded simply providing a PHR may have limited impact on empowerment and other outcomes without additional education or clinical intervention designed to increase PHR use.

Riippa et al.^30^ conducted a study in a Finnish public primary care setting. Study participants were at least 18 years old, with at least two treatable health conditions assessed by a health professional, bank identifiers (i.e., electronic credentials for online authentication provided by their bank), access to the Internet, and were willing and able, both according to themselves and to a healthcare professional, to engage in using the portal. The intervention group ended higher on the 13-item Short Form PAM (PAM-13) than the control group, but the difference was not statistically significant on raw figures (*p* = 0.9) or when using propensity score matching.

O’Leary et al.^31^ conducted a study in a large academic hospital in Chicago, Illinois and found that hospital in-patients given a PHR system (*n* = 100) scored higher on the PAM-13 compared to a control group (*n* = 102), but this was not statistically significant on a univariate analysis (*p* = 0.5), nor when adjusting for age (*p* = 0.4). This study did not explicitly describe the control group intervention.

Druss et al.^32^ evaluated the effect of a PHR on the quality of medical care in a community mental health setting in the US. Although having a PHR resulted in significantly improved quality of medical care, increased use of medical services among patients, there was not significant difference between experimental and control groups.

Creber et al.^35^ conducted a three-arm RCT study (usual care; iPad with general internet access; or iPad with access to the personalised inpatient portal) in two cardiac medical–surgical units at an academic medical centre in New York City in 2014–2017. The patient portal contained clinical information from the hospital's EHR and useful information about the patient's care team, a list of administered medications and educational content and functionality to communicate comments and questions to the care team. There were no significant differences in PAM scores among the three groups in unadjusted (*p* = 0.39) or adjusted (*p* = 0.42) analyses. Participants in the portal group who used the portal more frequently (two or more total logins) were not found to have higher activation (*p* = 0.11).

Carroll et al.^34^ conducted a pragmatic RCT in eight practices in New York and two in New Jersey serving patients living with HIV (PLWH). The objective of the study was to evaluate the effect of a multimodal self-management program, consisting of access to the *URHealth* app (electronic PHR [ePHR]) loaded onto an iPod with internet connection, combined with a targeted, peer-led and group-based intervention for PLWH. The intervention consisted of six 90-min group training sessions and a single 20- to 30-min individual pre-visit coaching session. The study primary measure was change in PAM. The study participants were at least 18 years old (mean age 52 in the intervention group and 51 in the control group) and with confirmed HIV status. Control group participants also received the iPod device after their follow-up evaluation was complete. The intervention group (*n* = 180) ended statistically significantly higher on the PAM-13 than the control group (*n* = 179), with a 2.8-unit improvement in patient activation. Patient activation improved at 12 months to 73.4 ± 1.13 and 70.5 ± 1.14 in the intervention and usual care groups, respectively (*p* = 0.027). The intervention was found to have a moderating effect on PAM level. The interaction between the intervention and PAM level was statistically significant among those in the lowest PAM quartile at baseline (*p* < 0.05). Notably, the intervention was associated with double the odds of improving one PAM level compared with control (odds ratio 1.96; 95% confidence interval 1.16–3.31).

### Meta-analysis

**Figure 2.**
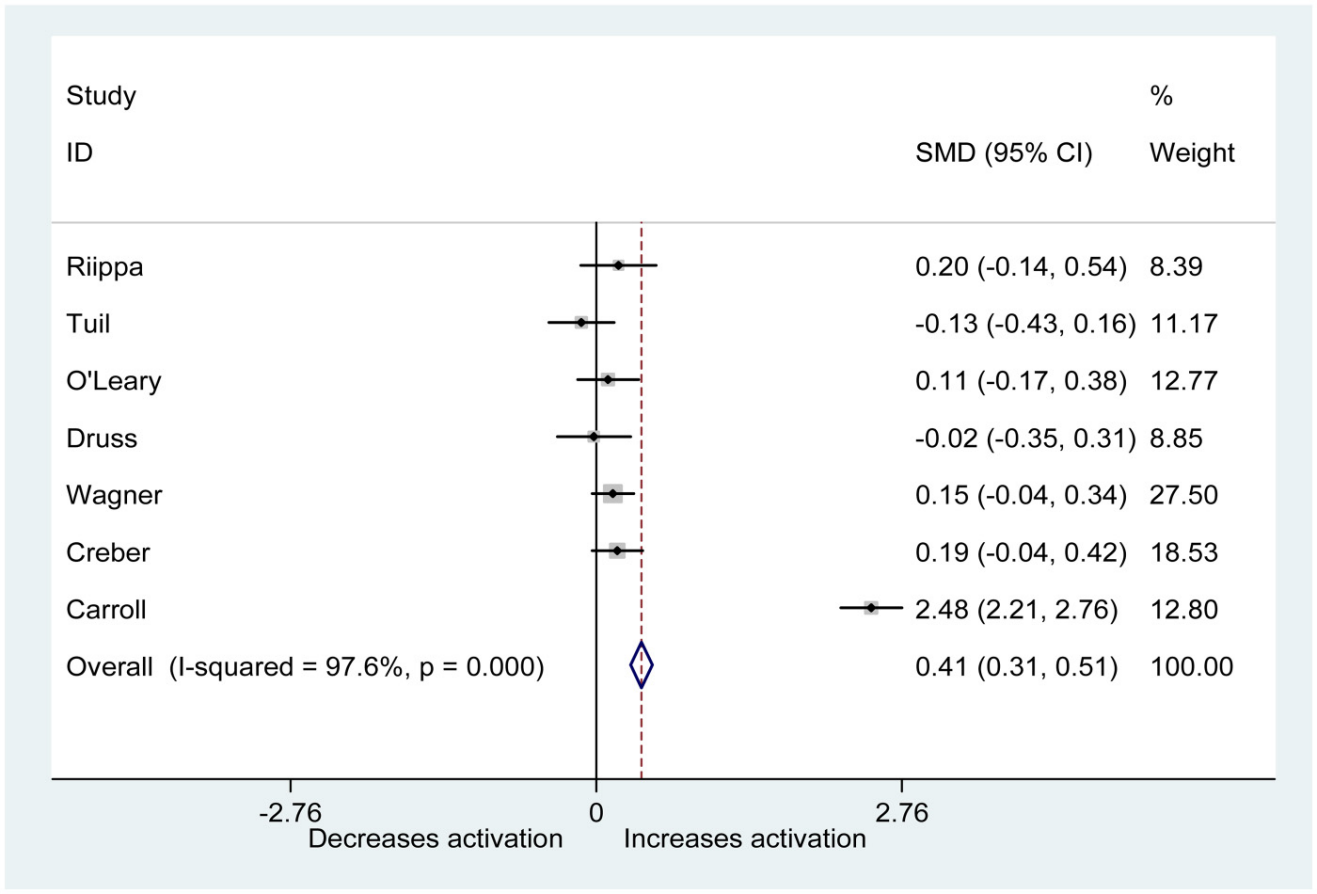
Forest plot showing effects of PHRs on patient activation.

A random effects meta-analysis was performed on seven of the included trials (Figure 2). Due to a lack of detail in the Earnest et al. study, data from this paper could not be extracted for the meta-analysis.

The quality assessment found all the studies to be of a reasonable and similar quality. The lowest scoring study was Earnest et al., which already had to be excluded from the meta-analysis. No analyses by quality level were thus conducted.

Wagner et al.^29^ give a mean in both groups at the end of study, but not a standard deviation. However, a baseline standard deviation is provided and we used this. The paper also gives a *p*-value for a comparison at end of study adjusting for background variables, including gender and literacy. This shows less difference than the raw numbers that we used in the meta-analysis. Wagner et al. did show a significant difference on another related outcome, PES.

The overall effect in a random effects meta-analysis is statistically significant (*z* = 8.12, *p* < 0.001). Heterogeneity is apparent: *I*^2^ = 98%, *χ*^2^(6) = 253.9, *p* < 0.0001 (Table 4). The effect seen in Carroll et al. is not consistent with the other studies, as is apparent from the forest plot (Figure 2).

**Table 4.** Meta-analysis of seven studies.

Study	SMD	95% confidence	Interval	% Weight
Rippa	0.201	−0.140	0.541	9.62
Druss	−0.021	−0.352	0.311	10.15
Carroll	2.485	2.209	2.760	12.80
Tuil	−0.134	−0.429	0.162	12.81
O’Leary	0.107	−0.169	0.383	14.64
Creber	0.191	−0.038	0.420	21.25
Wagner	0.151	−0.037	0.339	31.54
I-V pooled SMD	0.409	0.310	0.507	100.00

Heterogeneity *χ*^2^(6) = 253.9, *p* < 0.001; *I*^2^ (variation in SMD attributable to heterogeneity) = 97.6%; TEST of SMD = 0; *z* = 8.12, *p* < 0.001.

In a meta-analysis of six studies excluding Carroll et al., there is no statistically significant difference between intervention groups using a PHR and control groups: *z* = 1.93, *p* = 0.054. However, the result does approach statistical significance. There is now no heterogeneity apparent: *I*^2^ = 0%, *χ*^2^(5) = 4.1, *p* = 0.5.

## Discussion

### Principal findings

We found eight studies, six RCTs and two quasi-experimental, directly assessing the effects of PHRs on patient activation and similar measures. The effect seen in the Carroll et al. study was markedly greater than in the others. Across the other seven studies, we failed to find a statistically significant difference in the activation and patient empowerment measures, either individually or when pooled in a meta-analysis, although the pooled result was close to the 5% cut-off. The study by Wagner et al. has the greatest weight (28%) in the meta-analysis. The paper itself concludes a slight positive effect, although our version of the analysis to allow a consistent meta-analysis does not reach statistical significance.

We note that there are substantial differences across all the studies included in the meta-analysis. Studies ranged from publication in 2004 (Tuil et al. and Earnest et al.) to 2019 (Creber et al. and Carroll et al.) and are on different patient populations. There are functional differences in the PHR systems in these studies and in the approach to how the health systems introduced PHRs to patients and engaged them in using PHRs. Although limited information is available on the functional differences between the systems, we note that PHRs in five studies (Riippa et al., Tuil et al., Wagner et al., Carroll et al. and Creber et al.) had access to health data and information as well as the ability to interact with clinicians via some form of messaging system. PHRs in the other two studies (O’Leary et al. and Druss et al.) only contained access to various forms of health data and health-related information.

The Carroll et al. study stands out. Without it, the evidence for an effect by PHRs on activation is very weak. Carroll et al. evaluated the effect of a PHR for people living with HIV. The intervention consisted of an ePHR loaded on an iPod as well as coaching and peer support to increase knowledge and confidence in managing their health and healthcare as well as skills and tools to facilitate self-management. The population in Carroll et al. study also had relatively high levels of baseline PAM and a high level of self-management, which is typical for HIV patients.^36^ This study showed a greater effect on patient activation among those with the lowest levels of baseline patient activation, which might partly reflect a ceiling effect.

### Limitations

During this systematic review, we found only a fairly small number of studies that met our search criteria that evaluated the impact of PHRs on patient activation or empowerment measures. Apart from Wagner et al., most studies were relatively small in size; a larger number of studies and studies on bigger samples would provide greater statistical power. While Carroll et al. found a large effect, more studies are needed to replicate such findings among non-HIV patients with low income and with low health technology used to assess impact and the cost-effectiveness of multimodal approaches to patient activation. There is also a question on how self-management can be better integrated with standard care such as interactions with clinicians.

When we began this review, our hypothesis was that the use of PHRs leads to higher activation and therefore to better clinical outcome. As the systematic review concluded that the evidence to support this hypothesis was not significant, perhaps future research needs to look at preceding factors to patient activation and empowerment such as patient's engagement, ability and willingness to use PHRS. Below is a proposed logical model that breaks down the process and impact into a smaller stages and maybe helpful to visualise the journey of using PHRs for better health outcomes.


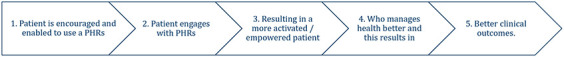


## Conclusion

Digitally enabling patients is a primary theme to many healthcare organisations and governmental initiatives. Central to these are patient access and interaction with their health records giving patients control over viewing, modifying and sharing their clinical records, and sharing the information between healthcare professionals, patients and carers.

The Carroll et al. study shows a notable shift in patient activation, and this is the study that pushed hard on patient engagement with the PHR. Literature review has demonstrated that patients’ interest to access and update their own health data is growing but the ability to use patient portals is often influenced by personal factors including age, education level, health literacy and health status.^2^ National PHR adoption strategy and healthcare providers’ endorsement appear to be some of the fundamental drivers in PHR adoption.^37^ As the patient-facing features of PHR portals are still evolving, redesigned care pathways to incorporate the use of PHR technology, where appropriate, will be fundamental in creating a sustainable environment for patient portals use. Adoption by patients and endorsement by providers will come when existing patient portal features align with patients’ and providers’ information needs and functionality.^38^

If patient health records are to become the norm, then there needs to be a better understanding of the factors that contribute to creating value and practical use.^30,31^ PHRs have the potential to impact on patient activation, but the effect does not appear to be consistent. How the PHR is introduced may be important to realising that effect. In Carroll et al. study, we observed that the PHR combined with coaching and training created a more significant impact on PAM but there may be also an opportunity to personalise the patient experience through better design of a PHR system.

Future research in these areas should focus on interventions that target and measure actual PHR usage, engagement in care and what modifiable factors contribute to meaningful PHR use by patients. This can give insights to how best improve PHR system design, better introduce systems to patients and support them in making the best use of PHRs for their healthcare. Further investigation is needed to see how certain approaches in personalisation could have a positive effect.

## Supplemental Material

sj-docx-1-dhj-10.1177_20552076251315295 - Supplemental material for A systematic review of the effect of personal health records on patient activation Supplemental material, sj-docx-1-dhj-10.1177_20552076251315295 for A systematic review of the effect of personal health records on patient activation by Irina Osovskaya, Ann Blandford and Henry WW Potts in DIGITAL HEALTH

## Appendix

**Notation**
ePHR: electronic personal health record
PAM: patient activation measure
PLWH: patients living with HIV
PES: patient empowerment scale
PHR: personal health record
PCHR: personally controlled health record
RCT: randomised controlled trial
SM: secure messaging

## References

[1] GOV.UK [Internet]. [cited 2024 May 27]. G7 patient access to health records: final report. Available from: https://www.gov.uk/government/publications/g7-health-track-digital-health-final-reports/g7-patient-access-to-health-records-final-report.

[2] Irizarry T Dabbs AD Curran CR . Patient portals and patient engagement: A state of the science review. J Med Internet Res [Internet] 2015; 17(6): e148. Available from: https://www.ncbi.nlm.nih.gov/pmc/articles/PMC4526960/. 10.2196/jmir.4255 PMC4526960 26104044

[3] Weitzman ER Kaci L Mandl KD . Acceptability of a personally controlled health record in a community-based setting: Implications for policy and design. J Med Internet Res 2009; 11: e14. 10.2196/jmir.1187 PMC2762802 19403467

[4] Tang PC Ash JS Bates DW , et al. Personal health records: Definitions, benefits, and strategies for overcoming barriers to adoption. J Am Med Inform Assoc JAMIA 2006; 13: 121–126. 16357345 10.1197/jamia.M2025 PMC1447551

[5] Nazi KM Hogan TP McInnes DK , et al. Evaluating patient access to electronic health records: Results from a survey of veterans. Med Care 2013; 51: S52–S56. 10.1097/MLR.0b013e31827808db 23407012

[6] Nøhr C Parv L Kink P , et al. Nationwide citizen access to their health data: Analysing and comparing experiences in Denmark, Estonia and Australia. BMC Health Serv Res 2017; 17: 534. 28784173 10.1186/s12913-017-2482-y PMC5547535

[7] Benefits of Personal Health Records - NHS Digital [Internet]. [cited 2020 Oct 4]. 2019. Available from: https://digital.nhs.uk/services/personal-health-records-adoption-service/personal-health-records-adoption-toolkit/benefits-of-personal-health-records.

[8] Davis Giardina T Menon S Parrish DE , et al. Patient access to medical records and healthcare outcomes: A systematic review. J Am Med Inform Assoc JAMIA 2014; 21: 737–741. 24154835 10.1136/amiajnl-2013-002239 PMC4078277

[9] Mold F Raleigh M Alharbi NS , et al. The impact of patient online access to computerized medical records and services on type 2 diabetes: Systematic review. J Med Internet Res 2018 Jul 6; 20(7): e235. 10.2196/jmir.7858 PMC6054706 29980499

[10] Han HR Gleason KT Sun CA , et al. Using patient portals to improve patient outcomes: Systematic review. JMIR Hum Factors 2019; 6: e15038. 10.2196/15038 PMC6940868 31855187

[11] de Lusignan S Mold F Sheikh A , et al. Patients’ online access to their electronic health records and linked online services: A systematic interpretative review. BMJ Open 2014; 4: e006021. 10.1136/bmjopen-2014-006021 PMC4158217 25200561

[12] Ammenwerth E Neyer S Hörbst A , et al. Adult patient access to electronic health records. Cochrane Database Syst Rev [Internet] 2021 [cited 2022 Dec 27]; 2(2): CD012707. Available from. https://www.cochranelibrary.com/cdsr/doi/10.1002/14651858.CD012707.pub2/full. 10.1002/14651858.CD012707.pub2 PMC8871105 33634854

[13] Engaging hospitalized patients with personalized health information: a randomized trial of an inpatient portal [Internet]. [cited 2020 Oct 18]. Available from: https://www.ncbi.nlm.nih.gov/pmc/articles/PMC6339515/pdf/ocy146.pdf 10.1093/jamia/ocy146 PMC6339515 30534990

[14] Supporting people to manage their health - Kings Fund.

[15] NHS England » Patient activation [Internet]. [cited 2020 Sep 21]. 2019. Available from: https://www.england.nhs.uk/personalisedcare/supported-self-management/patient-activation/.

[16] Fumagalli LP Radaelli G Lettieri E , et al. Patient empowerment and its neighbours: Clarifying the boundaries and their mutual relationships. Health Policy 2015; 119: 384–394. 25467286 10.1016/j.healthpol.2014.10.017

[17] MobiHealthNews [Internet]. [cited 2022 Aug 10]. 2019. Making sense of patient engagement, activation and empowerment. Available from: https://www.mobihealthnews.com/content/making-sense-patient-engagement-activation-and-empowerment.

[18] Irizarry T Shoemake J Nilsen ML . Patient portals as a tool for health care engagement: A mixed-method study of older adults with varying levels of health literacy and prior patient portal use. PubMed [Internet]. 2017 [cited 2020 Oct 5]. Available from: https://pubmed.ncbi.nlm.nih.gov/28360022/10.2196/jmir.7099 PMC5391436 28360022

[19] Toscos T Daley C Heral L , et al. Impact of electronic personal health record use on engagement and intermediate health outcomes among cardiac patients: A quasi-experimental study. J Am Med Inform Assoc 2016; 23: 119–128. 26912538 10.1093/jamia/ocv164 PMC7814924

[20] Impact of electronic personal health record use on engagement and intermediate health outcomes among cardiac patients: A quasi-experimental study. 10.1093/jamia/ocv164 PMC7814924 26912538

[21] Murray E Hekler EB Andersson G , et al. Evaluating digital health interventions: Key questions and approaches. Am J Prev Med 2016; 51: 843–851. 27745684 10.1016/j.amepre.2016.06.008 PMC5324832

[22] ScienceDirect [Internet]. Topics randomized controlled trial—An overview. [cited 2024 May 27]. Available from: https://www.sciencedirect.com/topics/neuroscience/randomized-controlled-trial

[23] Harris AD McGregor JC Perencevich EN , et al. The use and interpretation of quasi-experimental studies in medical informatics. J Am Med Inform Assoc JAMIA 2006; 13: 16–23. 16221933 10.1197/jamia.M1749 PMC1380192

[24] Martin J . © Joanna Briggs Institute 2017 Critical Appraisal Checklist for Randomized Controlled Trials. 2017; Available from: https://jbi.global/sites/default/files/2019-05/JBI_RCTs_Appraisal_tool2017_0.pdf.

[25] Martin J . © Joanna Briggs Institute 2017 Critical Appraisal Checklist for Quasi-Experimental Studies. 2017; Available from: https://jbi.global/sites/default/files/2019-05/JBI_Quasi-Experimental_Appraisal_Tool2017_0.pdf.

[26] Liberati A Altman DG Tetzlaff J , et al. The PRISMA statement for reporting systematic reviews and meta-analyses of studies that evaluate health care interventions: Explanation and elaboration. PLoS Med 2009; 6: e1000100. 10.1371/journal.pmed.1000100 PMC2707010 19621070

[27] Earnest MA Ross SE Wittevrongel L , et al. Use of a patient-accessible electronic medical record in a practice for congestive heart failure: Patient and physician experiences. J Am Med Inform Assoc 2004; 11: 410–417. 15187074 10.1197/jamia.M1479 PMC516248

[28] Tuil WS Verhaak CM Braat DDM , et al. Empowering patients undergoing in vitro fertilization by providing Internet access to medical data. Fertil Steril 2007; 88: 361–368. 17416366 10.1016/j.fertnstert.2006.11.197

[29] Wagner PJ Dias J Howard S , et al. Personal health records and hypertension control: A randomized trial. J Am Med Inform Assoc JAMIA 2012; 19: 626–634. 22234404 10.1136/amiajnl-2011-000349 PMC3384099

[30] Riippa I Linna M Rönkkö I . A patient portal with electronic messaging: Controlled before-and-after study. J Med Internet Res 2015; 17: e250. 10.2196/jmir.4487 PMC4642411 26553595

[31] O’Leary KJ Lohman ME Culver E , et al. The effect of tablet computers with a mobile patient portal application on hospitalized patients’ knowledge and activation. J Am Med Inform Assoc 2016; 23: 159–165. 26078412 10.1093/jamia/ocv058 PMC7814920

[32] Druss BG Ji X Glick G , et al. Randomized trial of an electronic personal health record for patients with serious mental illnesses. Am J Psychiatry 2014; 171: 360–368. 24435025 10.1176/appi.ajp.2013.13070913

[33] Masterson Creber R Prey J Ryan B , et al. Engaging hospitalized patients in clinical care: Study protocol for a pragmatic randomized controlled trial. Contemp Clin Trials 2016; 47: 165–171. 26795675 10.1016/j.cct.2016.01.005 PMC4818160

[34] Carroll JK Tobin JN Luque A , et al. Get Ready and Empowered About Treatment’ (GREAT) study: A pragmatic randomized controlled trial of activation in persons living with HIV. J Gen Intern Med 2019; 34: 1782–1789. 31240605 10.1007/s11606-019-05102-7 PMC6712153

[35] Masterson Creber RM Grossman LV Ryan B , et al. Engaging hospitalized patients with personalized health information: A randomized trial of an inpatient portal. J Am Med Inform Assoc 2019; 26: 115–123. 30534990 10.1093/jamia/ocy146 PMC6339515

[36] Kendall CE Shoemaker ES Crowe L , et al. Patient activation among people living with HIV: A cross-sectional comparative analysis with people living with diabetes mellitus. AIDS Care 2018; 30: 1444–1451. 29792355 10.1080/09540121.2018.1469723

[37] RCP London [Internet]. Personal health record (PHR)—Landscape review. Available from: https://www.rcplondon.ac.uk/projects/outputs/personal-health-record-phr-landscape-review. 2016.

[38] Irizarry T Shoemake J Nilsen ML , et al. Patient portals as a tool for health care engagement: A mixed-method study of older adults with varying levels of health literacy and prior patient portal use. J Med Internet Res 2017; 19: 1–1. 10.2196/jmir.7099 PMC5391436 28360022

